# Supplementary material for: COZOID: contact zone identifier for visual analysis of protein-protein interactions
Source: BMC Bioinformatics. 2018 Apr 6;19:125. doi: 10.1186/s12859-018-2113-6 (PMC5889581; doi:10.1186/s12859-018-2113-6)
Supplement: Supplementary file 2 — Software build. Executable binary file of the software tool. (ZIP 118,784 kb) [file 12859_2018_2113_MOESM2_ESM.zip › cozoid/java/docs/beansbinding-1.2.1-doc.zip!/org/jdesktop/beansbinding/PropertyResolutionException.html]

PropertyResolutionException (Beans Binding)


---


|  |  |  |
| --- | --- | --- |
| **Overview** \| **Package** \| **Class** \| **Use** \| **Tree** \| **Deprecated** \| **Index** \| **Help** | |  |
| **PREV CLASS**   **NEXT CLASS** | **FRAMES**    **NO FRAMES**     **All Classes** |  |
| SUMMARY: NESTED \| FIELD \| CONSTR \| METHOD | DETAIL: FIELD \| CONSTR \| METHOD |  |


---


## org.jdesktop.beansbinding Class PropertyResolutionException

```
java.lang.Object
  java.lang.Throwable
      java.lang.Exception
          java.lang.RuntimeException
              org.jdesktop.beansbinding.PropertyResolutionException
```

**All Implemented Interfaces:**: java.io.Serializable

---

``` public class PropertyResolutionException extends java.lang.RuntimeException ```

`PropertyResolutionExceptions` can be thrown at various points in
the life cycle of a `Property`. Any time a `Property`
encounters an exception in resolving a property, a
`PropertyResolutionException` can be thrown. For example, if a
`BeanProperty` encounters an exception while trying to resolve
the "foo" property of an object via reflection, the exception is
wrapped in a `PropertyResolutionException` and is re-thrown.

**See Also:**: Serialized Form

---

**Constructor Summary**

`PropertyResolutionException(java.lang.String message)`
  
          Creates a `PropertyResolutionException` with the given message.

`PropertyResolutionException(java.lang.String message,
java.lang.Exception reason)`
  
          Creates a `PropertyResolutionException` with the given message
and cause.


**Method Summary**

**Methods inherited from class java.lang.Throwable**

`fillInStackTrace, getCause, getLocalizedMessage, getMessage, getStackTrace, initCause, printStackTrace, printStackTrace, printStackTrace, setStackTrace, toString`

**Methods inherited from class java.lang.Object**

`clone, equals, finalize, getClass, hashCode, notify, notifyAll, wait, wait, wait`

**Constructor Detail**

### PropertyResolutionException

```
public PropertyResolutionException(java.lang.String message)
```

:   Creates a `PropertyResolutionException` with the given message.

    **Parameters:**: `message` - the exception's message

---


### PropertyResolutionException

```
public PropertyResolutionException(java.lang.String message,
                                   java.lang.Exception reason)
```

:   Creates a `PropertyResolutionException` with the given message
    and cause.

    **Parameters:**: `message` - the exception's message: `reason` - the original exception that caused this exception to be thrown


---


|  |  |  |
| --- | --- | --- |
| **Overview** \| **Package** \| **Class** \| **Use** \| **Tree** \| **Deprecated** \| **Index** \| **Help** | |  |
| **PREV CLASS**   **NEXT CLASS** | **FRAMES**    **NO FRAMES**     **All Classes** |  |
| SUMMARY: NESTED \| FIELD \| CONSTR \| METHOD | DETAIL: FIELD \| CONSTR \| METHOD |  |


---
